# Prospective association between leukocyte telomere length and mortality: findings from the Black Women's Experiences Living with Lupus (BeWELL) Study

**DOI:** 10.1093/cei/uxag052

**Published:** 2026-08-27

**Authors:** David H Chae, Connor D Martz, Amani M Nuru-Jeter, Tiffany Yip, Jue Lin, Eloisa Bonfá, Elissa S Epel, Kenneth Saag, Cristina M Lanata, Sofia Nadgauda, Jesus Ramirez-Valles, Jinoos Yazdany

**Affiliations:** Department of Social, Behavioral, and Population Sciences, Celia Scott Weatherhead School of Public Health and Tropical Medicine, Tulane University, New Orleans, LA, USA; Department of Population Health Sciences, University of Central Florida, College of Medicine, Orlando, FL, USA; Department of Community Health Sciences, School of Public Health, University of California, Berkeley, Berkeley, CA, USA; Department of Psychology, Fordham University, New York City, NY, USA; Department of Biochemistry and Biophysics, University of California, San Francisco, San Francisco, CA, USA; Hospital das Clinicas HCFMUSP, Faculdade de Medicina, Rheumatology Division, Universidade de Sao Pãulo, Sao Pãulo, Brazil; Department of Psychiatry and Behavioral Sciences, University of California, San Francisco, San Francisco, CA, USA; Department of Medicine, The University of Alabama at Birmingham, Birmingham, AL, USA; National Human Genome Research Institute, National Institutes of Health, Bethesda, MD, USA; Department of Social, Behavioral, and Population Sciences, Celia Scott Weatherhead School of Public Health and Tropical Medicine, Tulane University, New Orleans, LA, USA; Department of Medicine, University of California, San Francisco, San Francisco, CA, USA; Department of Medicine, University of California, San Francisco, San Francisco, CA, USA

**Keywords:** systemic lupus erythematosus, leukocyte telomere length, Black/African American women, survival analysis

## Abstract

Leukocyte telomere length (LTL) is a biomarker of replicative history of cells and has been posited to be an indicator of biological aging. LTL may yield insight into immunomodulatory disorders, including systemic lupus erythematosus (SLE), an immune-mediated inflammatory disease that disproportionately affects Black/African American women. This study examined the association between LTL and mortality among 422 Black/African American women in the Black Women's Experiences Living with Lupus (BeWELL) Study. Participants were recruited from metropolitan Atlanta, Georgia, between April 2015 and May 2017 and followed for a mean of 1.98 years (SD = 0.38). LTL was measured as the telomere-to-single copy gene ratio (T/S). Mortality was assessed prospectively. Cox proportional hazards regression models adjusting for sociodemographic, health, and disease characteristics were specified. A total of 19 participants died during the follow-up period. Adjusting for demographic, socioeconomic, and health-related covariates, longer LTL was associated with lower risk of mortality (hazard ratio = 0.13, 95% confidence interval = 0.02, 0.83, *P* = 0.03). This study is the first to report an association between LTL and subsequent mortality among Black/African American women with SLE. Findings may be particularly relevant for this population, which has been shown to experience severe disease consequences. Future research may further explore LTL in mechanistic studies of SLE and assess the utility of LTL for monitoring disease progression and outcomes.

## Introduction

Systemic lupus erythematosus (SLE) is a chronic autoimmune disease that in the US disproportionately impacts Black/African American women of childbearing age [1, 2]. Approximately 9 out of 10 cases of SLE are among women; Black/African American women have 2–3 times the prevalence compared to White women [3] and also experience more severe disease consequences, including irreversible organ damage and mortality [4–6]. One study reported that among incident cases of SLE, mortality was more than 10 years earlier among Black/African Americans compared to Whites (51.8 years vs. 64.4 years of age) [7]. Although risk factors for severity and progression of SLE are multi-level and multi-factorial, ranging from macrosocial to individual biological risk factors, research suggests that they are mediated by immune and inflammatory mechanisms central to the etiopathogenesis of the disease [8–10].

Telomere length (TL) may be a meaningful biomarker to study in the context of immunomodulatory and inflammation-mediated conditions [11–14]. Telomeres are repetitive sequences of DNA at the ends of chromosomes that protect against DNA degradation and support chromosomal stability [15, 16]. In eukaryotes, the DNA sequences at the terminal end of the lagging strand are lost during replication and shorten over time. Accordingly, TL has been posited to be a marker of replicative history and aging at the cell level [17–19]. Critically short telomeres result in cellular senescence and subsequent release of inflammatory molecules [11, 20]. In turn, telomeres shorten more rapidly under conditions of oxidative stress and inflammation [21–23]. Leukocyte telomere length (LTL) specifically has emerged as a marker of general systemic aging among humans and has been associated with several aging-related health outcomes as well as mortality [24, 25].

Research has generally shown that telomeres are shorter among those with autoimmune disease compared to the general population, and that shorter TL is associated with indicators of greater disease severity [20, 26–28]. People with SLE specifically have been found to have shorter telomeres compared to the general population [29, 30]. Among those with SLE, research indicates that Black/African Americans experience faster telomere shortening [31, 32]. A handful of studies have examined associations between TL and measures of disease severity among people with SLE, suggesting that elevated inflammation that is characteristic of SLE may result in dysregulation of the telomere maintenance system [33, 34]. However, to our knowledge, no studies have examined the association between TL and health outcomes prospectively in this population.

The extant literature suggests that TL may be an important biomarker of disease severity to consider in the context of SLE. In this study, we examine the association between LTL and incident mortality among Black/African American women with SLE using data from the Black Women's Experiences Living with Lupus (BeWELL) Study.

## Methods and materials

### Participants

Protocols and procedures of the BeWELL Study have been previously described [4, 35]. Briefly, 438 self-identified Black/African American women with SLE were recruited from metropolitan Atlanta, Georgia. Participants were enrolled between April 2015 and May 2017 from the broader Georgians Organized Against Lupus (GOAL) cohort [36], which was largely drawn from the Georgia Lupus Registry—a Centers for Disease Control and Prevention population-based registry with supplemental recruitment from the lupus clinic at a large public hospital and rheumatology practices. All participants in the GOAL cohort had a validated diagnosis of SLE, meeting ≥4 classification criteria from the American College of Rheumatology, or 3 with a diagnosis by a board-certified rheumatologist.

The BeWELL Study consisted of three main study visits—baseline, 12 months, and 24 months. The final study visit was conducted in February 2019. Main study visits were primarily conducted in person at the Division of Rheumatology at Emory University School of Medicine, which involved interviewer- and self-administered survey questions and a brief exam including collection of dried blood spots (DBSs) from a finger prick. In addition to study visits, two short interim surveys between waves were collected at 6 and 18 months. All study protocols and procedures were approved by the Institutional Review Board of Emory University.

### Measures

#### Leukocyte telomere length

Procedures for LTL assays in the BeWELL Study have been previously described [30]. LTL was assayed from genomic DNA extracted from DBS collected at baseline using QIAmp DNA investigator kit (QIAGEN; catalog # 56504) [37]. Six 3-mm punches from each sample yielded an average of 175 ng ± 97 ng in 50 μl elution buffer. Assays were performed in duplicate for each sample and measured as the telomere-to-single copy gene ratio (T/S). In instances where T/S varied by more than 7%, the sample was run a third time, and the two that were closest in value were retained. The average coefficient of variation for this study was 2.3% ± 1.6%.

#### Mortality

Mortality was assessed prospectively from the date of baseline enrollment through the study conclusion date and was confirmed by documentation from clinical notes, obituaries, or notices received from family members. Time-to-event was calculated as the interval between the participant's baseline enrollment date and date of death. For those who were censored (i.e. nondeceased), follow-up time was calculated as the interval between their baseline date and their date of last contact (i.e. the date of their final survey). Because all censored participants completed at least one follow-up assessment, follow-up time was calculated precisely based on specific dates without midpoint assumptions. Both the total length of follow-up and time-to-event were calculated in days, then converted to years by dividing the total days by 365.25.

#### Covariates

All covariates included in analyses were assessed at baseline. Sociodemographic characteristics were self-report measures of: age in years; years since SLE diagnosis; relationship status (1 = married, married-like or other romantic relationship, 2 = widowed, divorced, or separated; 3 = single, never married); insurance status (1 = private, 2 = public, 3 = uninsured); educational attainment (1 = high school or less, 2 = some college or associate's degree, 3 = college degree, 4 = graduate degree); and ratio of household income to the poverty threshold (based on the number of adults and children under 18 years of age) [38]. Indicators of disease severity were: organ damage assessed using the Brief Index of Lupus Damage (BILD), which includes a range of comorbid conditions (e.g. cardiometabolic, malignancies, musculoskeletal) [39]; and disease activity measured using the Systemic Lupus Activity Questionnaire (SLAQ), which assesses symptoms of lupus in the past 3 months [40]. SLE medication use was measured dichotomously for the following: hydroxychloroquine; steroids; and immunosuppressants.

#### Analysis plan

We excluded four participants whose death occurred after their participation in the study (subsequent to Wave 3) because mortality status was not systematically assessed for all participants. An additional 12 participants with missing data on any study variables were also excluded from the analytic sample. All analyses were conducted using the remaining complete cases (n = 422) using SAS 9.4 (SAS Institute Inc., Cary, NC). Kaplan–Meier survival analysis was used to visually estimate the unadjusted probability of survival over time by baseline LTL tertiles. Cox proportional hazards regression models were specified to estimate the hazard ratio (HR) and 95% confidence interval (CI) for the association between baseline LTL (modeled continuously) and mortality.

## Results

**Figure 1:**
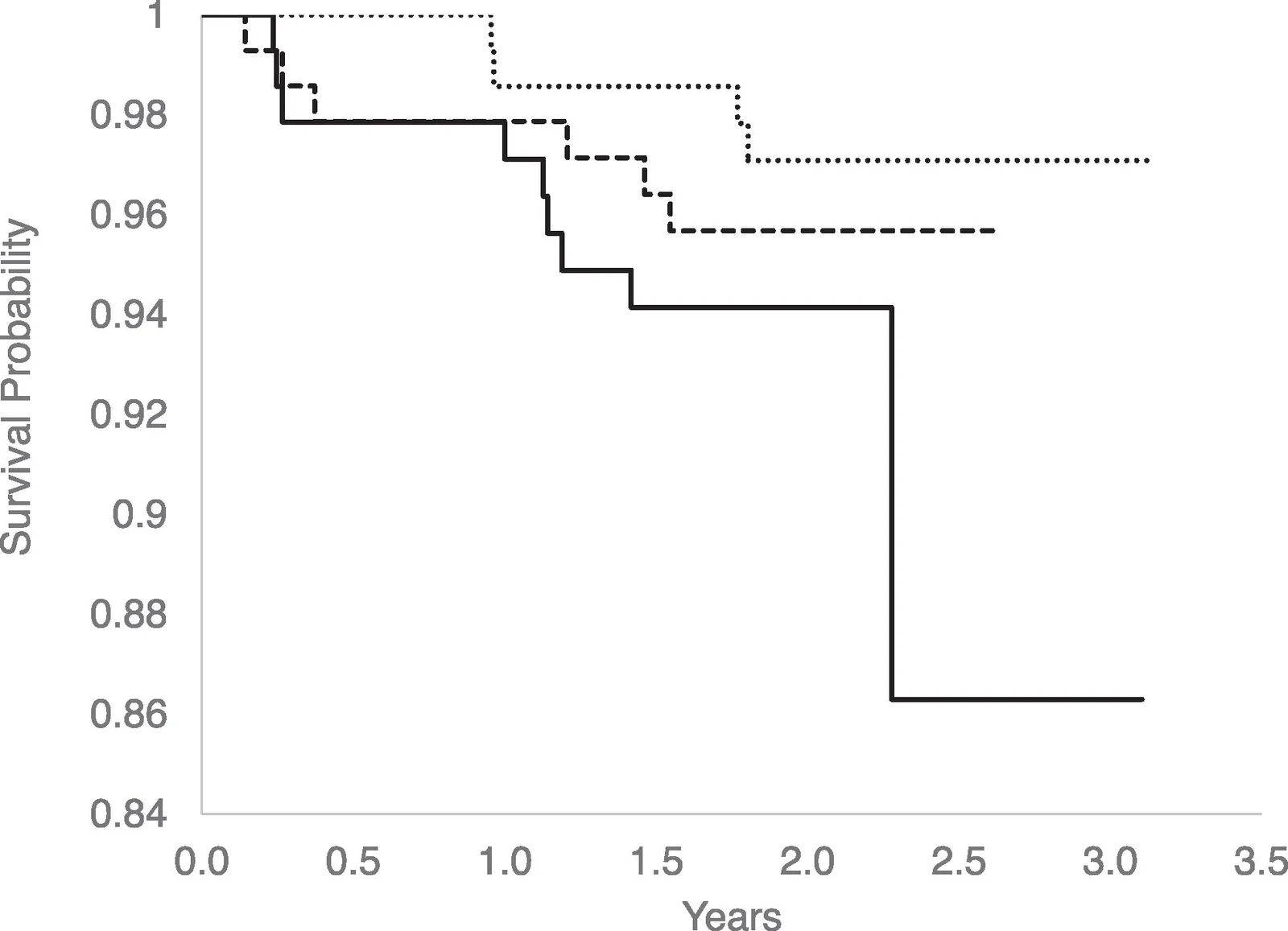
Kaplan–Meier curve of survival probability over time by leukocyte telomere length (LTL) tertiles in the Black Women's Experiences Living with Lupus (BeWELL) study (*n* = 422; 2015–2019). 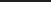 Short LTL, 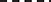 Medium LTL, 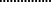 Long LTL.

A total of 19 participants died during the follow-up period. Deceased participants had significantly shorter mean baseline LTL (mean = 1.25, standard deviation [SD] = 0.30) compared with nondeceased participants (mean = 1.40, SD = 0.29; t = 2.17, *P* = 0.04). The Kaplan—Meier curve illustrating the unadjusted probability of survival by LTL tertile is shown in Fig. 1. Additional participant characteristics among those deceased and nondeceased are shown in Table 1.

**Table 1:** Sociodemographic characteristics of participants in the Black Women's Experiences Living with Lupus (BeWELL) study (*n* = 422; 2015–2019).

	Mean (SD) or *n* (%)		
	Nondeceased (*n* = 403)	Deceased (*n* = 19)	Total (*n* = 422)
Leukocyte telomere length, mean (SD)*	1.40 (0.29)	1.25 (0.30)	1.40 (0.29)
Age, mean (SD)	47.01 (12.19)	46.07 (14.63)	46.97 (12.29)
Disease duration in years, mean (SD)	15.86 (10.47)	18.06 (10.31)	15.96 (10.46)
Relationship status, *n* (%)^†^			
Married, romantic relationship	215 (53.35)	6 (31.58)	221 (52.37)
Widowed, separated, divorced	87 (21.59)	4 (21.05)	91 (21.56)
Single	101 (25.06)	9 (47.37)	110 (26.07)
Insurance status, *n* (%)*			
Private	153 (37.97)	2 (10.53)	155 (36.73)
Public	204 (50.62)	15 (78.95)	219 (51.90)
Uninsured	46 (11.41)	2 (10.53)	48 (11.37)
Education, *n* (%)			
High school or less	103 (25.56)	9 (47.37)	112 (26.54)
Some college or associate's	183 (45.41)	6 (31.58)	189 (44.79)
College degree	69 (17.12)	1 (5.26)	70 (16.59)
Graduate degree	48 (11.91)	3 (15.79)	51 (12.09)
Income-to-poverty ratio, mean (SD)***	2.07 (1.70)	1.19 (0.71)	2.03 (1.68)
Organ damage, mean (SD)^†^	2.51 (2.54)	3.47 (2.12)	2.55 (2.53)
Disease activity, mean (SD)	15.08 (8.11)	16.05 (6.14)	15.13 (8.03)
Steroids, *n* (%)*			
No	184 (45.66)	4 (21.05)	188 (44.55)
Yes	219 (54.34)	15 (78.95)	234 (55.45)
Hydroxychloroquine, *n* (%)			
No	107 (26.55)	8 (42.11)	115 (27.25)
Yes	296 (73.45)	11 (57.89)	307 (72.75)
Immunosuppressants, *n* (%)^†^			
No	227 (56.33)	7 (36.84)	234 (55.45)
Yes	176 (43.67)	12 (63.16)	188 (44.55)
Length of follow-up, mean (SD)***	2.03 (0.30)	1.02 (0.62)	1.98 (0.38)

^†^ *P* < 0.10 ^*^  *P* < 0.05 **P < 0.01 ***P < 0.001

Results from Cox proportional hazards regression models are shown in Table 2. Adjusting for age and years since SLE diagnosis, there was a significant inverse relationship between LTL and mortality, with longer LTL being associated with lower mortality risk. Each standard deviation increase in LTL was associated with an 90% lower risk of mortality (Model 1: HR = 0.10, 95% CI = 0.01,0.71, *P* = 0.02). This relationship remained statistically significant after controlling for additional demographic characteristics (Model 2: HR = 0.11, 95% CI = 0.02, 0.74; *P* = 0.02), and further adjustment for health-related factors, including the BILD and SLAQ (Model 3: HR = 0.13, 95% CI = 0.02, 0.83; *P* = 0.03).

**Table 2:** Cox proportional hazards analysis of leukocyte telomere length and mortality in the Black Women's Experiences Living with Lupus (BeWELL) study (*n* = 422; 2015–2019).

	Hazard ratio (95% confidence interval)		
	Model 1	Model 2	Model 3
Leukocyte telomere length	0.10 (0.01, 0.71)*	0.11 (0.02, 0.74)*	0.13 (0.02, 0.83)*
Age	0.97 (0.92, 1.01)	0.97 (0.92, 1.03)	0.97 (0.92, 1.02)
Years since diagnosis	1.03 (0.98, 1.08)	1.03 (0.98, 1.08)	1.04 (0.98, 1.09)
Relationship status (Ref = married, romantic relationship)			
Widowed, separated, divorced		1.43 (0.37, 5.46)	1.34 (0.33, 5.51)
Single		3.20 (1.10, 9.27)*	3.52 (1.12, 11.04)*
Insurance status (ref = private)			
Public		2.85 (0.54, 15.18)	2.28 (0.42, 12.29)
Uninsured		1.88 (0.22, 16.00)	1.67 (0.19, 14.74)
Education (ref = graduate degree)			
High school or less		0.73 (0.17, 3.03)	1.57 (0.32, 7.61)
Some college or associate's		0.28 (0.07, 1.19)^†^	0.46 (0.10, 2.08)
College degree		0.22 (0.02, 2.11)	0.32 (0.03, 3.42)
Income-to-poverty ratio		0.70 (0.38, 1.31)	0.74 (0.38, 1.42)
Organ damage			1.06 (0.86, 1.31)
Disease activity			0.98 (0.92, 1.04)
Steroids: yes vs. no			2.82 (0.82, 9.63)^†^
Hydroxychloroquine: yes vs. no			0.37 (0.12, 1.12)^†^
Immunosuppressants: yes vs. no			1.95 (0.67, 5.72)

^†^ *P* < 0.10 ^*^  *P* < 0.05 **P < 0.01 ***P < 0.001.

Sensitivity analyses were conducted including participants whose date of death was recorded after the completion date of their final Wave 3 survey (*n* = 4), first, coding them as deceased and calculating their follow-up time using their date of mortality; and next, coding them as nondeceased and recording their follow-up time using the date of their final assessment. In both scenarios, findings were substantively unchanged, with longer LTL being associated with lower mortality risk (HR = 0.17, 95% CI = 0.03, 0.98, *P* = 0.048; and HR = 0.12, 95% CI = 0.02, 0.82, *P* = 0.03, respectively).

## Discussion

To our knowledge, this is the first study to evaluate the relationship between LTL and mortality among Black/African American women with SLE. We found that shorter LTL is associated with higher mortality risk, even after adjustment for potential confounders, including disease duration, socioeconomic factors, and indicators of disease severity. Our findings resonate with those of previous research suggesting that LTL may be an informative biomarker in autoimmune rheumatic diseases more broadly [13, 14, 20, 41].

LTL may reflect cumulative inflammatory burden and immune senescence relevant to SLE pathophysiology. LTL may also propagate immune dysregulation and a heightened inflammatory state through replicative senescence. Senescent cells produce a senescence-associated secretory phenotype, which activates immune pathways and promotes inflammation [42, 43]. In addition, chronic inflammation causes damage to surrounding tissues; when dead cells are not efficiently cleared, secondary necrotic bodies contribute to a pro-inflammatory state [44, 45]. Consistent with these observations, people with SLE have been found to have shorter LTL than the general population [29]. Furthermore, LTL has been shown to shorten more rapidly among Black/African Americans with SLE compared to their White counterparts [14, 46], which may reflect racial disparities in disease outcomes [36, 47]. In the context of SLE, LTL may signal biological tolls associated with the accumulation of inflammation and general immune system aging [20].

The findings of this study should be considered given several caveats. The BeWELL Study specifically recruited Black/African American women from the Atlanta, Georgia metropolitan area, and findings from this research may not be generalizable to other racial or gender groups, or those in other geographic areas. Potential SLE-related alterations in leukocyte subpopulations were not assessed, which may partially influence LTL measurements [48–50]. The relatively short follow-up period precludes assessment with respect to long-term mortality risk. Accordingly, it is possible that short LTL reflects more imminent risk resulting from severe illness burden. In addition, the observational nature of the data limits conclusions regarding causality and causal direction. For instance, although we adjusted for baseline indicators of disease severity, it is possible that advanced disease or pre-terminal physiological decline preceded LTL shortening. Analytic concerns include the limited number of events, which restricted our ability to conduct stratified analyses or test interactions by potential moderators. Multivariable models including numerous correlated covariates may have also been subject to overfitting. Residual confounding by other unmeasured factors is also a possible concern. Future large, well-powered research that longitudinally assesses relationships between LTL and SLE outcomes across the lifespan and among a broader range of people affected by SLE is warranted.

## Conclusion

This study provides novel preliminary evidence supporting the relevance of LTL as a biomarker associated with SLE outcomes. Despite limitations, our findings are consistent with the existing literature on immunosenescence in SLE. Findings are specifically pertinent for Black/African American women, a group that has been found to be at heightened risk for severe disease and adverse consequences. This formative study suggests that LTL may merit inclusion in future research as a sentinel biomarker in basic and applied SLE research, including mechanistic, prognostic, and translational studies of SLE, in addition to a broader set of serologies and established panels.

## Data Availability

The datasets used and/or analyzed during the current study are available from the corresponding author upon reasonable request.

## Abbreviations

BeWELL: Black Women's Experiences Living with Lupus
BILD: Brief Index of Lupus Damage
CI: confidence interval
DBS: dried blood spots
GOAL: Georgians Organized Against Lupus
HR: hazard ratio
LTL: leukocyte telomere length
SLAQ: Systemic Lupus Activity Questionnaire
SLE: systemic lupus erythematosus
TL: telomere length
T/S: telomere-to-single copy gene ratio
